# Traditional medicine and its contributions to science, health equity and sustainability

**DOI:** 10.2471/BLT.25.295061

**Published:** 2025-11-01

**Authors:** Shyama Kuruvilla, Geetha Krishnan Gopalakrishna, Pillai, Sungchol Kim, Lori McDougall, Goh Cheng Soon, Motlalepula Matsabisa, Sylvie Briand

**Affiliations:** a Global Traditional Medicine Centre, World Health Organization, Interim Office, ITRA Campus, Jamnagar – 361008, Gujarat, India.; b Traditional and Complementary Medicine Division, Ministry of Health, Kuala Lumpur, Malaysia.; c University of Free State, Free State, South Africa.; d Science Division, World Health Organization, Geneva, Switzerland.

The World Health Organization’s (WHO) new *Global traditional medicine strategy 2025–2034* aims to advance the contribution of evidence-based traditional, complementary and integrative medicine to the highest attainable standard of health and well-being.^1^^,^^2^ In support of this goal, in December 2025, the second WHO Global Summit on Traditional Medicine will convene global leaders under the theme of the science and practice of restoring balance, underscoring the importance of implementing the traditional medicine strategy.^3^

This theme issue of the *Bulletin of the World Health Organization* supports the summit’s agenda, by exploring how the integration of traditional medicine can enrich health systems, promote UHC and support inclusive, sustainable development. Doing so requires anchoring traditional medicine in a transformative scientific frame, ensuring its safety and efficacy, and redefining health as achieving a dynamic balance of self, society and ecosystems that promotes well-being. The local and cultural foundation of traditional medicine should be preserved, and the fundamental values of equity in access and rights of Indigenous communities remain at the centre of the global strategy. The articles in this issue detail pathways via responsible scaling, cultural respect, knowledge protection and equitable benefit-sharing. 

One article describes how Chapter 26 of the 11th International Statistical Classification of Diseases and Related Health Problems enables the coding of traditional medicine disorders and patterns alongside biomedical diagnoses, filling long-standing data gaps and making traditional medicine visible in health information systems.^4^ At the population level, another article demonstrates how national health surveys can be refined to better capture who uses traditional medicine and why.^5^

To facilitate systems integration, a model for strengthening the traditional medicine workforce is proposed.^6^ A systematic review identifies enablers and barriers to integration into primary health care in low- and middle-income countries, from governance and financing to education and standards.^7^

Traditional medicine is the primary or preferred care for billions of people worldwide. Analysis of 71 nationally representative surveys shows its widespread use for hypertension, diabetes and hypercholesterolemia, often alongside conventional care.^8^ The clinical potential is considerable, exemplified by the Zang-Fu theory-guided framework for cancer supportive care, which links organ-based imbalances to standardized interventions.^9^ Interest in mental health and well-being is also growing, supported by an expanding evidence base for practices such as mindfulness and a global shift towards whole-health systems.^10^ However, challenges remain; for instance, while acupuncture is recommended for migraine, many guidelines show methodological and procedural gaps.^11^

Health systems integration requires safety as a foundation. One article calls for a global hub for medicinal plant safety that integrates traditional knowledge with modern science, complemented by a respect for cultural and local ecosystems integrity.^12^ Another underscores that for Indigenous Peoples, access to land, language and data sovereignty is inseparable from sustaining their medicine systems and intergenerational knowledge transmission. This recognition expands the notion of evidence, which is generated in trials and surveys but also grounded in rights, traditions and stewardship.^13^ Safety is thus pharmacological, ecological and cultural, based on both science and sovereignty.

Traditional medicine is increasingly used in the health, wellness and bioeconomy sectors. Nonetheless, an analysis revealed that less than 1% of global health research funding is dedicated to traditional medicine, an inequity that undermines efforts to build the required evidence base.^14^ Decolonizing research paradigms, protecting Indigenous knowledge systems, and ensuring equitable benefit-sharing are also critical. The 2024 World Intellectual Property Organization treaty on intellectual property, genetic resources and associated traditional knowledge provides a milestone for fairer innovation. The success of this treaty depends on effective national implementation and substantive engagement of Indigenous Peoples and all traditional medicine stakeholders.^15^

Innovation can accelerate traditional medicine progress and scale, but only if guided by ethics, equity and ecological sensitivity. One article describes how artificial intelligence (AI) can enhance diagnostics and personalize treatment but warns of risks around biases and data security. Such caution is particularly relevant given the threat of automated biopiracy, where AI could systematically mine traditional knowledge without consent.^16^

Traditional medicine is more than a collection of therapies; it represents a worldview in which health is harmony within and between individuals, communities and ecosystems. Restoring this balance is a scientific, rights-based and sustainability imperative.
